# Multiple change point detection and validation in autoregressive time series data

**DOI:** 10.1007/s00362-020-01198-w

**Published:** 2020-07-13

**Authors:** Lijing Ma, Andrew J. Grant, Georgy Sofronov

**Affiliations:** 1 Department of Mathematics and Statistics, Macquarie University, Sydney, Australia; 2 MRC Biostatistics Unit, University of Cambridge, Cambridge, UK

**Keywords:** Changepoint detection, Autoregressive time series, Likelihood ratio scan statistics, Multiple testing problems

## Abstract

It is quite common that the structure of a time series changes abruptly. Identifying these change points and describing the model structure in the segments between these change points is of interest. In this paper, time series data is modelled assuming each segment is an autoregressive time series with possibly different autoregressive parameters. This is achieved using two main steps. The first step is to use a likelihood ratio scan based estimation technique to identify these potential change points to segment the time series. Once these potential change points are identified, modified parametric spectral discrimination tests are used to validate the proposed segments. A numerical study is conducted to demonstrate the performance of the proposed method across various scenarios and compared against other contemporary techniques.

## 1 Introduction

The statistical properties of time series data, such as mean and variance or the coefficients of the regression model, may change abruptly at unknown time points. Identifying those unknown time points is referred to as change point detection or time series segmentation. The change point problem was first considered by Page (1954) and Page (1955) for quality control. Since then, the topic has been explored theoretically and computationally in the field of statistics and computer science, and has been applied to economics (Bai and Perron 2003; Bai 2010), finance (Aue and Horváth 2013; Andreou and Ghysels 2009), and biology (Olshen et al. 2004; Niu and Zhang 2012). Furthermore, see the recent survey papers by Jandhyala et al. (2013), Aminikhanghahi and Cook (2017) and Truong et al. (2020) for the development of univariate or multivariate time series segmentation methods.

There are essentially two types of approaches for detecting unknown change points under a parametric design: the model selection method and the traditional hypothesis testing method. Model selection or exact segmentation methods generally include two elements, a cost function and an optimization algorithm. The computational complexity depends on the complexity of data and the number of change points. In contrast, the approximate segmentation methods have significantly less computational cost when there are more change points. Here, we follow in the direction of the approximate segmentation methods.

One popular representation of the approximate segmentation methods is the binary segmentation (BS) family of methods. The core idea is that BS tests if there is a change point in the process at each step or iteration (see Fryzlewicz 2014 for a detailed description). BS has gained huge popularity due to the minor computational cost and its user-friendliness. However, the method may ignore change points if the length of the segment is relatively short. Hence, Olshen et al. (2004) further improved the BS algorithm, and proposed the circular BS (CBS) method. Fryzlewicz (2014) proposed the wild BS (WBS) approach to detect the number and locations of changes in a piecewise stationary model when the values of the parameters change. Another representation of the approximate segmentation methods is bottom-up segmentation, which is less explored than the BS algorithm (we recommend the paper by Keogh et al. 2001 for further details). Bottom-up segmentation is also easy to apply: the first step is to obtain a sequence of overestimated change points; the second step is to eliminate the falsely-detected ones.

However, both the BS algorithm and the bottom-up method may suffer from the multiple testing problem. Eichinger et al. (2018) mentions in regards to the BS algorithm that “it can be difficult to interpret the results in terms of significance due to the multiple testing involved”. Thus, Fryzlewicz (2014) added a randomized segment selection step to the BS method. Li et al. (2016) proposed multiscale change point segmentation with controlled false discovery rate (FDR) based on multiscale statistics considered by Frick et al. (2014) for inferring the changes in the mean of an independent sequence of random variables. Cao and Wu (2015) developed a large scale multiple testing procedure for data with clustered signals. The earlier references that introduced FDR for multiple change point detection include Niu and Zhang (2012) and Hao et al. (2013), which are motivated by genome data. Hitherto, only a small amount of literature attempts to address this issue. When the observations are dependent, detecting multiple change points is quite a difficult task, especially in the case of autoregressive processes. Davis et al. (1995) studied the asymptotic behavior of the likelihood ratio statistic in testing if a change point has occurred in the mean, the auto-covariance structure or the order of an autoregressive process. Later on, Davis et al. (2006) estimated all the parameters of a piecewise stationary autoregressive process by using a genetic algorithm to optimize an information criterion as objective function. Hušková et al. (2007) firstly derived the limiting behavior of various max-type test statistics under the hypothesis of whether there is an autocorrelation coefficient change in an autoregressive time series, then compares the asymptotic results of these test statistics with corresponding resampling procedures in the paper of Hušková et al. (2008). Peštová and Pešta (2017) developed a method based on the ratio type statistic to test at most one possible regression parameter change in an AR(1) series. Chakar et al. (2017) proposed a robust approach for estimating change points in the mean of an AR(1) process. Korkas and Fryzlewicz (2017) upgraded the WBS algorithm by applying a locally stationary wavelet process for estimating change points in the second-order structure of a piecewise stationary time series model. Yau and Zhao (2016) proposed a likelihood ratio scan method (LRSM) to estimate change points in piecewise stationary processes.

In this paper, we develop a new Multiple Comparisons Procedure for a Multiple Change Point Problem (MCP-MCP, or MCP2 for short), to estimate the number and locations of change points in a piecewise stationary autoregressive model. The procedure includes three simple steps: the first step is to apply the likelihood ratio scan statistics by Yau and Zhao (2016) to obtain a set of potentially overestimated change points; the second step is to use the spectral discrimination procedure developed by Grant and Quinn (2017) to eliminate possibly falsely discovered change points; the third step is to use a classic controlling FDR procedure and an adjusted p-value Bonferroni procedure to address the multiple testing issue. Our work is mainly inspired by Yau and Zhao (2016) and Korkas and Fryzlewicz (2017) and, to the best of our knowledge, is the first paper to address the multiple testing issue taking the dependency into account as a bottom-up segmentation method.

As indicated by Mercurio and Spokoiny (2004), it is highly risky to treat non-stationary data as though they are from a stationary process when making predictions and forecasting. Therefore, the estimation accuracy tends to be very important and the exact properties of estimates need careful attention. In our simulation study, we focus on the correct estimated number and locations of change points. The structure of the paper is as follows. In Sect. 2, we provide the details of the MCP2 method. In Sect. 3, through extensive simulation experiments and in Sect. 4, through two real data examples, we evaluate the performance of the MCP2, LRSM and WBS methods. Lastly, we conclude the paper in Sect. 5 with discussion and comments on future research.

## 2 A multiple comparisons procedure for change point detection

### 2.1 Non-stationary time series segmentation as a multiple testing problem

We start this section by demonstrating the autoregression process segmentation problem, and how it can be viewed as a multiple hypothesis testing problem. Let *x*_1_, *x*_2_,..., *x*_*T*_ be a sequence of an autoregression process, with *q* the unknown number of change points and *k*_1_, *k*_2_,..., *k*_*q*_ their respective unknown positions, where 1 < *k*_1_ < *k*_2_ < ··· < *k*_*q*_ < *T*. The autoregression process with multiple change points is illustrated as below

$$x_{t}=\{\begin{array}{ll} \beta_{0}^{\left(1\right)}+\beta_{1}^{\left(1\right)}x_{t-1}+\cdots +\beta_{p_{1}}^{\left(1\right)}x_{t-p_{1}}+\varepsilon_{t}^{\left(1\right)}, & t=1,\ldots ,k_{1}\\ \beta_{0}^{\left(2\right)}+\beta_{1}^{\left(2\right)}x_{t-1}+\cdots +\beta_{p_{2}}^{\left(2\right)}x_{t-p_{2}}+\varepsilon_{t}^{\left(2\right)}, & t=k_{1}+1,\ldots ,k_{2}\\ \cdots & \\ \beta_{0}^{\left(q+1\right)}+\beta_{1}^{\left(q+1\right)}x_{t-1}+\cdots +\beta_{p_{q+1}}^{\left(q+1\right)}x_{t-p_{q+1}}+\varepsilon_{t}^{\left(q+1\right)}, & t=k_{q}+1,\ldots ,T \end{array}$$

where $\varepsilon_{t}\sim i.i.d.N\left(0,\sigma_{t}^{2}\right)$ and each segment is a stationary autoregression of order *p* (AR(p)) and independent of each other. This problem can be expressed as a classical single hypothesis testing problem, as follows. Letting *θ*_*t*_ be the parameters that generate the data at each time point, *t* = 1,..., *k*_*q*_,..., *T*,

(1) $$\begin{array}{l} H_{0}:\theta_{1}=\cdots =\theta_{k_{q}+1}=\cdots =\theta_{T}\\ H_{1}:\theta_{1}=\cdots =\theta_{k_{1}}\neq \theta_{k_{1}+1}=\cdots =\theta_{k_{2}}\neq \cdots \neq \theta_{k_{q}+1}=\cdots =\theta_{T} \end{array}$$

If *H*_1_ is supported, the data are split into *q* + 1 segments, (*x*_1_, *x*_2_,..., *x*_*k*_1__), (*x*_*k*_1_+1_, *x*_*k*_1_+2_,..., *x*_*k*_2__),..., (*x*_*k*_*q*_+1_, *x*_*k*_*q*_+2_,..., *x*_*T*_), with different generating parameters for each segment denoted by $\theta_{i}:=\left(p_{i},\beta_{p_{i}}^{\left(i\right)},\sigma^{2^{\left(i\right)}}\right)$, *i* = 1,..., *q* + 1.

The ambitious objective is to estimate the number of change points *q*, the location vector *k* (*k*_1_, *k*_2_, ···, *k*_*q*_) and the parameters for each segment *θ*_*i*_. It is not practical to achieve this objective through the aforementioned single hypothesis testing framework, hence we decompose (1) to multiple hypothesis tests

(2) $$\begin{array}{l} H_{0}\left(i\right):\theta_{k_{i-1}+1:k_{i}}=\theta_{k_{i}+1:k_{i+1}}\\ H_{1}\left(i\right):\theta_{k_{i-1}+1:k_{i}}\neq \theta_{k_{i}+1:k_{i+1}} \end{array}$$

for *i* = 1,..., *q*. Since we assume that each segment is an independent time series, (2) can be viewed as a multiple testing problem by determining whether two adjacent segments (*x*_*k*_*i*−1_+1_, *x*_*k*_*i*−1_+2_,..., *x*_*k*_*i*__) and (*x*_*k*_*i*_+1_, *x*_*k*_*i*_+2_,..., *x*_*k*_*i*_+1_) have been generated by the same underlying stochastic process. We use a parametric spectral discrimination approach to solve this problem.

### 2.2 Change points exploration by using scan statistics

In Sect. 2.1, we did not define the range of *q*, which could be any value between 0 and *T*. Therefore, as the first step, a possibly overestimated set of change points will be estimated by using the likelihood ratio scan statistics proposed by Yau and Zhao (2016). A brief introduction is given in this section.

For a window radius *h* we define a corresponding scanning window *R*_*t*_ (*h*) and observations as

$$\begin{array}{l} R_{t}\left(h\right)=t-h+1,\ldots ,t+h\\ x_{R_{f}\left(h\right)}=x_{t-h+1},\ldots ,x_{t+h} \end{array}$$

The likelihood ratio scan statistics is then

$$LS_{h}\left(t\right)=\frac{1}{h}L_{t-h+1,\ldots ,t}\left(t,{\hat{\theta }}_{1}\right)+\frac{1}{h}L_{t+1,\ldots ,t+h}\left(t,{\hat{\theta }}_{2}\right)-\frac{2}{h}L_{t-h+1,\ldots ,t+h}\left(t,\hat{\theta }\right)$$

where $L\left(\theta \right)=\sum_{t=1}^{T}\log f_{\theta }\left(x_{t}|x_{t-1},\ldots ,x_{t-p}\right)$

By scanning the observed time series data, a sequence of *L S*_*h*_ (*t*) will be obtained at *t* = *h*, *h* + 1, …, *T* – *h*. If *h* meets certain criteria, at most one change point outputs in *R*_*t*_ (*h*), and if there is a change at *t*, then *L S*_*h*_ (*t*) tends to be large. Hence, a set of potential change points *k̂* (*k*_1_, *k*_2_,..., *k*_*q*_) will be obtained after the scanning process.

### 2.3 A likelihood ratio test for comparing time series

Given a set of estimated change points, we then apply a modified version of the parametric spectral discrimination test proposed by Grant and Quinn (2017) to test if the adjacent segments are from the same autoregressive process. We fit the autoregressive models

$$\begin{array}{l} x_{t}+\beta_{x,1}x_{t-1}+\ldots +\beta_{x,p_{x}}x_{t-j}=\varepsilon_{t}\\ y_{t}+\beta_{y,1}y_{t-1}+\ldots +\beta_{y,p_{y}}y_{t-j}=u_{t} \end{array}$$

to two adjacent segments of lengths *T*_1_ and *T*_2_, respectively, where {*ε*_*t*_} and {*u*_*t*_} are independent processes with zero mean and variances $\sigma_{\varepsilon }^{2}$ and $\sigma_{u}^{2}$, respectively. Although the test is developed as though *ε*_*t*_ and *u*_*t*_ are i.i.d and Gaussian, the asymptotic distribution of the test statistic holds under much weaker conditions (Grant 2018). Note that we are also assuming that the processes have zero mean, and in practice the time series are mean-corrected before analysis. That is, we do not consider a shift in mean between segments to constitute a change point, but rather consider only changes in the second-order properties. The hypothesis test is

$$\begin{array}{l} H_{0}:\beta_{X,j}=\beta_{Y,j}\;\text{for}\;\text{all}\;j,\;\sigma_{\varepsilon }^{2}=\sigma_{u}^{2}\\ H_{A}:\;\text{Not}\;H_{0} \end{array}$$

Under the null hypothesis, the underlying processes share the same autocovariance structure, or, in other words, have the same spectral density (hence the term spectral discrimination tests). In order to compute the likelihood ratio statistic, we need the maximum likelihood estimators of the parameters under both *H*_0_ and *H*_*A*_. Under *H*_*A*_, the processes are independent and the parameters can be estimated separately using, for example, the Levinson–Durbin algorithm (Levinson 1947; Durbin 1960). For a given order *p*, the algorithm computes the estimators

$$\hat{\beta }P=-{\hat{\Gamma }}_{p}^{-1}{\hat{\gamma }}^{p},$$

$${\hat{\sigma }}_{p}^{2}=\hat{\gamma }\left(0\right)+{\left({\hat{\gamma }}^{p}\right)}^{\prime }{\hat{\beta }}^{p},$$

where

$${\hat{\beta }}^{p}={\left[\beta_{1}\cdots \beta_{p}\right]}^{\prime },\;{\hat{\gamma }}^{p}={\left[\gamma \left(1\right)\cdots \gamma \left(p\right)\right]}^{\prime },\;\hat{\gamma }\left(j\right)=\frac{1}{T}\sum_{t=j}^{T-1}x_{t}x_{t-j},$$

*T* is the sample size and *Γ̂*_*p*_ is the *p* × *p* matrix with (*i*, *j*)th entry given by *γ̂* (|*i* – *j*|). These estimators are the solutions to the Yule–Walker equations, and represent method of moment estimators of the model parameters. Asymptotically, they are equivalent to the maximum likelihood estimators under Gaussianity. Under *H*_0_, for *j* 0,..., *p*, we define

$$c\left(j\right)=\frac{1}{T_{1}+T_{2}}\left(\sum_{t=j}^{T_{1}-1}x_{t}x_{t-j}+\sum_{t=j}^{T_{2}-1}y_{t}y_{t-j}\right).$$

Replacing *γ̂* (*j*) by *c* (*j*) in the Levinson–Durbin algorithm gives estimators for the common parameters. The test statistic is

(3) $$\Lambda =T_{1}\log\left(\frac{{\hat{\sigma }}_{0}^{2}}{{\hat{\sigma }}_{\varepsilon ;A}^{2}}\right)+T_{2}\log\left(\frac{{\hat{\sigma }}_{0}^{2}}{{\hat{\sigma }}_{u;A}^{2}}\right),$$

where ${\hat{\sigma }}_{\varepsilon ;A}^{2}$ and ${\hat{\sigma }}_{u;A}^{2}$ are the estimators of $\sigma_{\varepsilon }^{2}$ and $\sigma_{u}^{2}$ under *H*_*A*_, and ${\hat{\sigma }}_{0}^{2}$ is the estimator of the common residual variance under *H*_0_. We reject *H*_0_ when *Λ* is greater than the 100 (1 – *α*)th percentile of the *χ* ^2^ distribution with *p*_*x*_ + *p*_*y*_ – *p* + 1 degrees of freedom.

Since the orders are unknown in practice, they can be estimated using, for example, an information criterion such as BIC. This is easily incorporated into the Levinson– Durbin algorithm. However, it was shown in Grant and Quinn (2017) that the test performs poorly when the underlying time series are not truly autoregressive. The proposed solution was to use autoregressive approximation by fixing the orders, under both *H*_0_ and *H*_*A*_, as *p*_*x*_ = *p*_*y*_ = *p* = ⌊(log *T*_min_)^*υ*^⌋, where *υ* > 1, *T*_min_ = min (*T*_1_, *T*_2_) and ⌊(log *T*_min_)^*υ*^⌋ is the integer component of (log *T*_min_)^*υ*^. The null hypothesis is then rejected when *Λ* is greater than the 100 (1 – *α*)th percentile of the *χ* ^2^ distribution with *p* + 1 degrees of freedom. The test then performs well even when the time series are not autoregressive, with the cost being some loss in power in the autoregressive case.

It is possible to adjust the test to consider a change in mean as a change point. In this case, the models we fit (using the fixed autoregressive order approach outlined above) are

$$\begin{array}{l} \left(x_{t}-\mu_{X}\right)+\beta_{x,1}\left(x_{t-1}-\mu_{X}\right)+\ldots +\beta_{x,p}\left(x_{t-j}-\mu_{X}\right)=\varepsilon_{t}\\ \left(y_{t}-\mu_{Y}\right)+\beta_{y,1}\left(y_{t-1}-\mu_{Y}\right)+\ldots +\beta_{y,p}\left(y_{t-j}-\mu_{Y}\right)=u_{t}, \end{array}$$

and the null hypothesis is

$$H_{0}^{*}:\beta_{X,j}=\beta_{Y,j}\;\text{for}\;\text{all}\;j,\;\sigma_{\varepsilon }^{2}=\sigma_{u}^{2}\;\mu_{X}=\mu_{Y}.$$

Letting

$${\hat{\mu }}_{X}=\frac{1}{T_{1}}\sum_{j=0}^{T_{1}-1}x_{t},\;{\hat{\mu }}_{Y}=\frac{1}{T_{2}}\sum_{j=0}^{T_{2}-1}Y_{t},\;\hat{\mu }=\frac{1}{T_{1}+T_{2}}\left(T_{1}{\hat{\mu }}_{X}+T_{2}{\hat{\mu }}_{Y}\right),$$

we replace *γ̂* (*j*) and *c* (*j*) by

$${\hat{\gamma }}^{*}\left(j\right)=\frac{1}{T}\sum_{t=j}^{T-1}\left(x_{t}-{\hat{\mu }}_{X}\right)\left(x_{t-j}-{\hat{\mu }}_{X}\right)$$

and

$$c^{*}\left(j\right)=\frac{1}{T_{1}+T_{2}}\left\{\sum_{t=j}^{T_{1}-1}\left(x_{t}-\hat{\mu }\right)|\left(x_{t-j}-\hat{\mu }\right)+\sum_{t=j}^{T_{2}-1}\left(y_{t}-\hat{\mu }\right)\left(y_{t-j}-\hat{\mu }\right)\right\}$$

respectively. The test statistic is then computed in the same way using parameter estimates from the Levinson–Durbin algorithm. The null hypothesis is rejected when *Λ* is greater than the 100 (1 – *α*)th percentile of the *χ* ^2^ distribution with *p* + 2 degrees of freedom.

### 2.4 Approaches for multiple hypothesis tests

Generally, for a single hypothesis test, we specify a Type I error, say 0.05, and make a conclusion based on the test statistic which meets this specification while giving the highest power. When multiple hypotheses are tested simultaneously, the probability of at least one incorrect “statistically significant” outcome is increased with as the number of independent tests increases, which may result in incorrect conclusions. Thus, it is necessary to evaluate the tests as a whole. Numerous procedures have been proposed for this multiple comparison problem. In this paper, we implement two classical procedures: Controlling the false discovery rate, proposed by Benjamini and Hochberg (1995) (BH); and the adjusted *p*-values approach of Wright (1992).

As per the previous subsection, we can obtain unadjusted p-values *p*_(1)_, *p*_(2)_,..., *p*_(*q*)_ corresponding to the multiple hypotheses considered in (2). Let *P*_(1)_ ≤ *P*_(2)_ ≤ ··· ≤ *P*_(*q*)_ be the ordered *p*_(1)_, *p*_(2)_,..., *p*_(*q*)_ from smallest to largest. The BH multiple-testing procedure is as follows.

For each *i* = 1, 2,..., *q*, if $P_{\left(i\right)}\leq \frac{i}{q}\alpha$

then reject all *H*_(*i*)_

k̂* = (*k*_1_, *k*_2_,..., *k*_*q*∗_) is the final estimates of change points.

Next, we adopt the adjusted *p*-values method by Bonferroni procedure as follows.

For each *i* = 1, 2,..., *q*, if *q* × *p*_(*i*)_ ≤ *α*

then reject all *H*_(*i*)_

k̂* = (*k*_1_, *k*_2_,..., *k*_*q*∗_) is the final estimates of change points.

## 3 Simulation study

### 3.1 Choice of scanning window

In this section, we use nine classic examples to compare the performance of the MCP2 method with methods from recent literature including the likelihood ratio scan method (LRSM) by Yau and Zhao (2016) and the wild binary segmentation method (WBS) by Korkas and Fryzlewicz (2017). Except for model G, the models used in the simulation study also were considered by Yau and Zhao (2016). For each model, we simulated 100 sequences. The first step of both the LRSM and MCP2 method is to obtain the possible change points by using likelihood ratio scan statistics, which involves the tuning parameter — scanning radius *h*. Theoretically, the LRSM requires *r* log(*T*)^2^ ≤ *h* ≤ *ml*_*k*_/2, where *ml*_*k*_ denotes the minimum length between the adjacent change points, *T* is the length of the time series, and *r* is specified by the user. The scanning radius *h* = max {50, 2 log(*T*)^2^} is suggested by Yau and Zhao (2016) as a rule-of-thumb. However, the LRSM may not be applicable when the *h* ≤ *ml*_*k*_/2 is violated, additionally, *h* ≤ *ml*_*k*_/2 criterion is not practical as the minimum distance of neighboring change points is unknown.

Hence, we implement a sensitivity analysis to study the optimal choice of *h* in the MCP2 method for each model, displayed by Table 1. In the table, % *N̂* = *N* denotes the the percentage that the estimated number of change points is the actual number. We also investigate average degrees of freedom of *χ* ^2^ distribution, as *p* = ⌊(log *T*_min_)^*υ*^⌋, the length of segment may be affected by the scanning window *h*.

We have tested multiple values of *h*, it is shown that the choice of *h* has an impact on the detection rate (% *N̂* = *N*). Optimal scanning window *h* can be selected based on two criteria. We first consider choosing the minimum value of *h* which gives the maximum detection rate (% *N̂* = *N*). Second, we select the value of *h* which is less than the first segment’s length. For example, the exact change point of Model D is located at 50, although the detection rate increased as *h* increased, the optimal value of *h* should be less than 50; otherwise, the actual change point is dismissed at the beginning. The optimal scanning window for each model is summarised in Table 2.

### 3.2 Comparison between methods

To measure the detection accuracy of the methods, we consider evaluating the estimated number of change points and the estimated locations separately. In this paper, we define the exact detection rate as the proportion that the estimated number of change points equals to the correct number of change points among 100 sequences, shown by % : *N̂* = *N* in Table 1. Table 3 summarises the performance in terms of estimated number of change-points for each model. In addition, we designed novel plots to display the distance between the actual and estimated locations of change points, which could help evaluate the detection accuracy on estimated locations.

In order to compare with LRSM, we used the same setting for both the LRSM and MCP2 method: *h* = 2 log(*T*)^2^, *ml*_*k*_ = 50 is set for Model A, B, C, G, and H; *h* log(*T*)^2^, *ml*_*k*_ = 25 is set for Model I; *h* = log(*T*)^2^, *ml*_*k*_ = 50 is set for Model D, E and F.

**Fig. 1:**
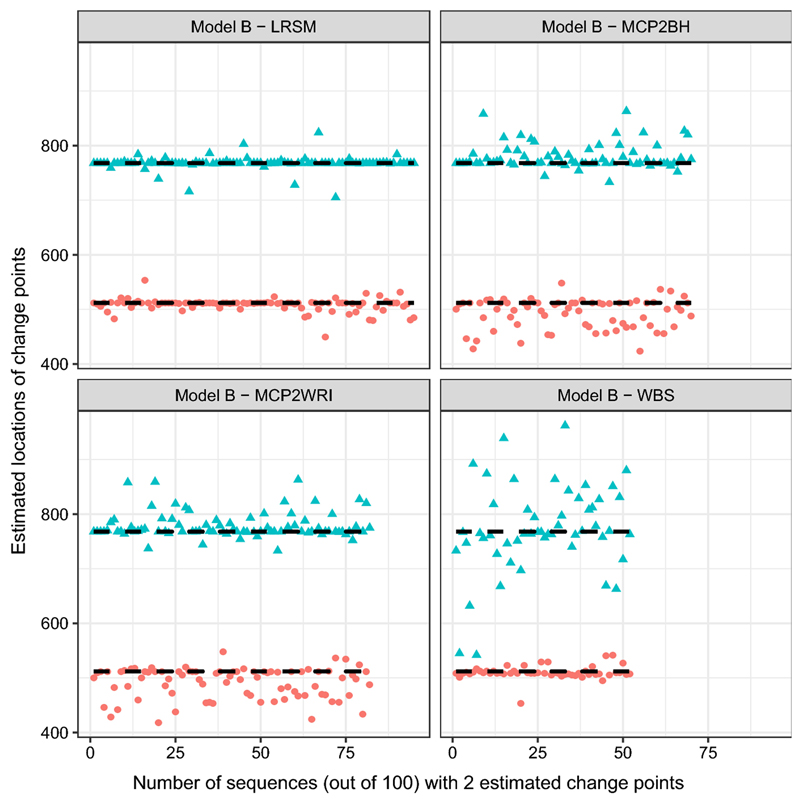
Plots of estimated locations of change points from different methods under model B. Horizontal line stands for the sequence of estimated changes only when the estimated number of change points equals to 2. The dashed black lines represent the true locations of change points, 512 and 768

**Fig. 2:**
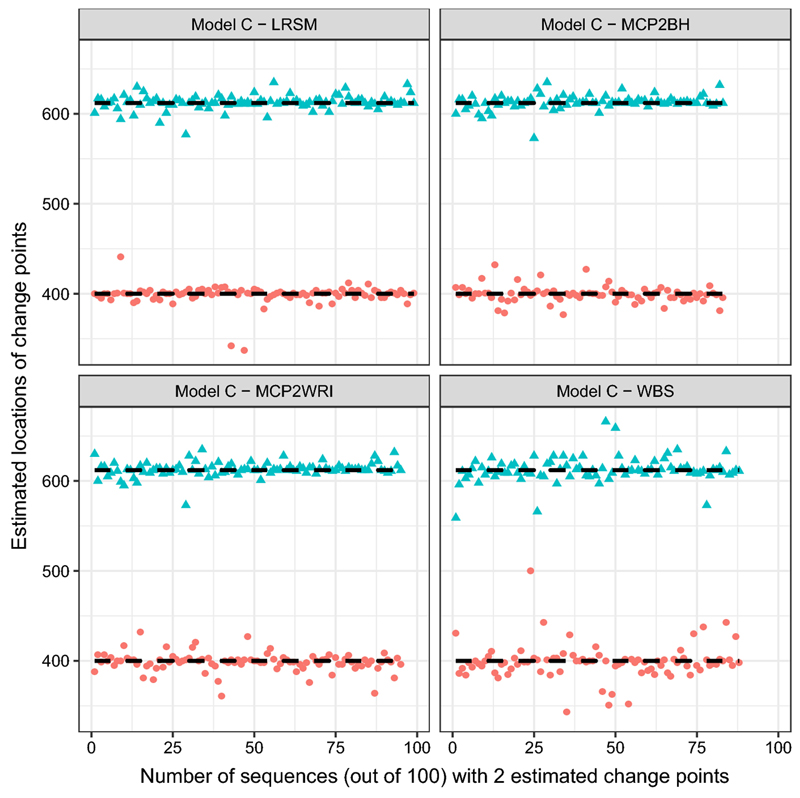
Plots of estimated locations of change points from different methods under model C. Horizontal line stands for the sequence of estimated changes only when the estimated number of change points equals to 2. The dashed black line represents the true locations of change points, 400 and 612

**Fig. 3:**
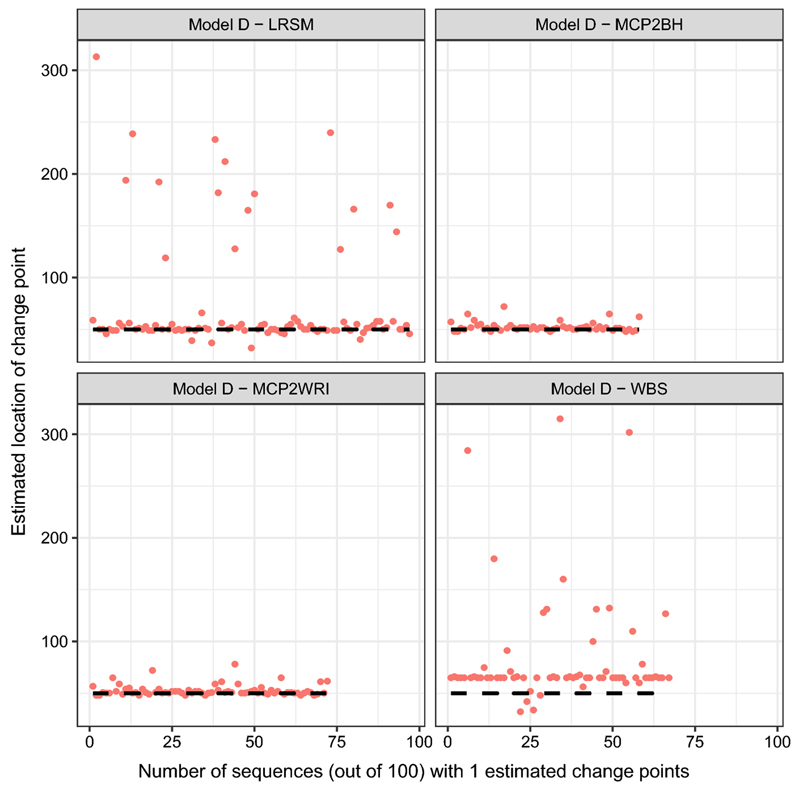
Plots of estimated locations of change points from different methods under model D. Horizontal line stands for the sequence of estimated changes only when the estimated number of change points equals to 1. The dashed black line represents the true location of change points at 50

**Fig. 4:**
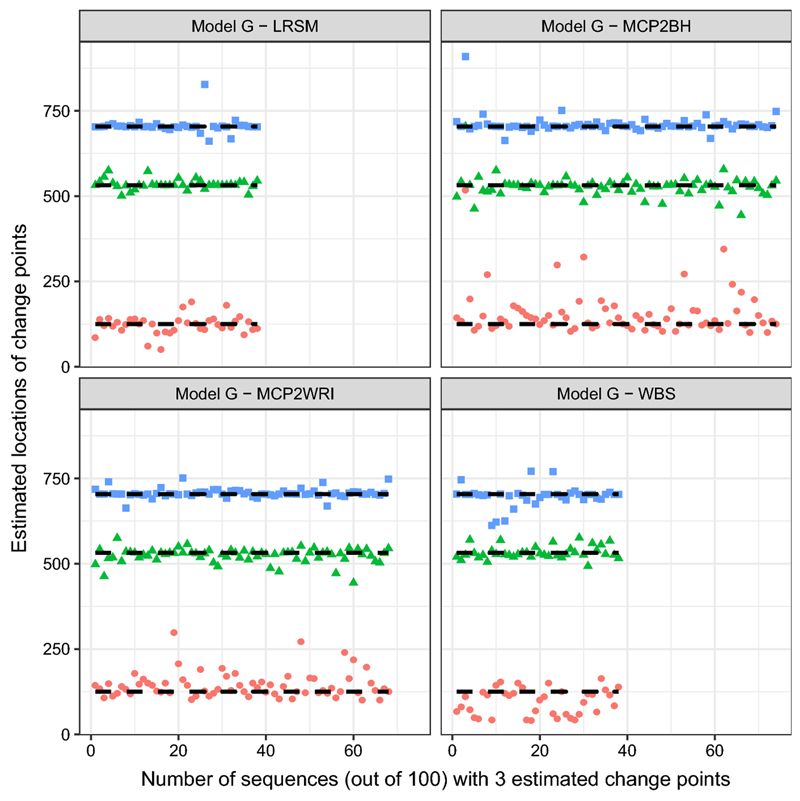
Plots of estimated locations of change points from different methods under model G. Horizontal line stands for the sequence of estimated changes only when the estimated number of change points equals to 3. The dashed black line represents the true locations of change points, 125, 532 and 704

**Fig. 5:**
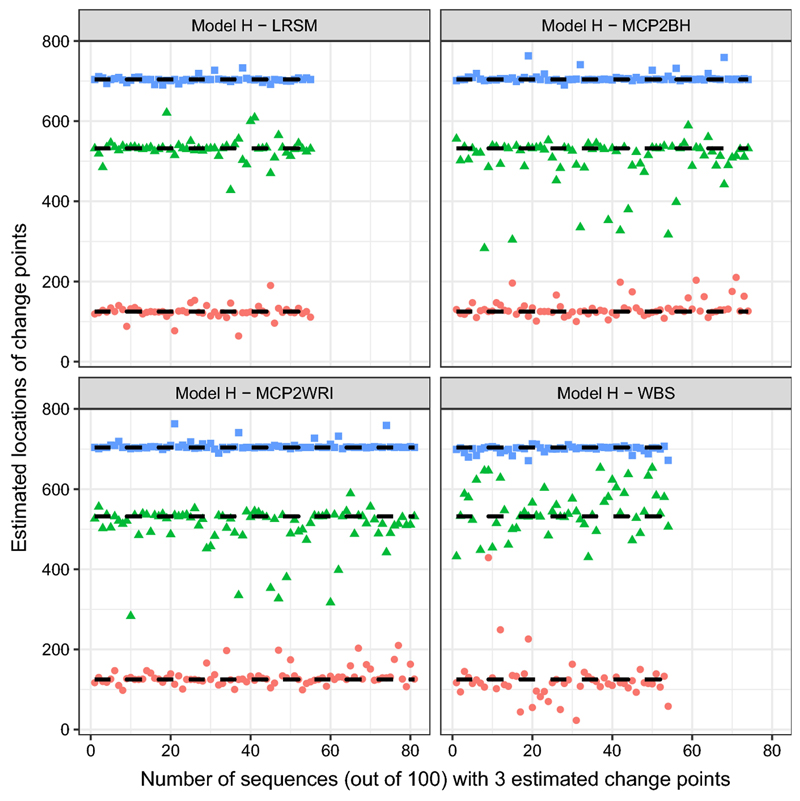
Plots of estimated locations of change points from different methods under model H. Horizontal line stands for the sequence of estimated changes only when the estimated number of change points equals to 3. The dashed black line represents the true locations of change points, 125, 532 and 704

(a) Model A: stationary AR(1) process with various *β* = − 0.7, − 0.1, 0.4, 0.7
  (4) $$x_{t}=\beta x_{t-1}+\varepsilon_{t},1\leq t\leq 1024$$
  We evaluate the performance of the methods via Model A that there is no change point. LRSM is overall perfect under model A, WBS is nearly perfect except the poor performance when *β* = –0.7. MCP2 method performs well and almost uniformly with various *β*, while tends to have over-segmentation problem, regardless of the value of *h*.
(b) Model B: piecewise stationary auto-regressive process
  (5) $$x_{t}=\{\begin{array}{ll} 0.9x_{t-1}+\varepsilon_{t} & 1\leq t\leq 512\\ 1.69x_{t-1}-0.81x_{t-2}+\varepsilon_{t}, & 513\leq t\leq 768\\ 1.32x_{t-1}-0.81x_{t-2}+\varepsilon_{t}, & 769\leq t\leq 1024 \end{array}$$
  From Table 3, it is clear that LRSM is outstanding over the others, WBS has the lowest accuracy rate and tends to overestimate the number of change points, and MCP2BH suffers from overestimation as well. Moreover, LRSM gives the most accurate estimated locations which can be seen by looking at Fig. 1. Estimated location of WBS spaced out around the true location 768, compared with the estimates at 512, it seems to lose the power to detect the second change-point, which may be the reason for overestimation. If we look at the setting of Model B, at the second location, the coefficients of the adjacent AR(2) segments are very close, which make it difficult to detect. Similarly, the estimated locations of MCP2 methods show mild variation at 512 and 768.
(c) Model C: piecewise stationary AR(1) process
  (6) $$x_{t}=\{\begin{array}{ll} 0.4x_{t-1}+\varepsilon_{t} & 1\leq t\leq 400\\ -0.6x_{t-1}+\varepsilon_{t}, & 401\leq t\leq 612\\ 0.5x_{t-1}+\varepsilon_{t}, & 613\leq t\leq 1024 \end{array}$$
  Comparing with model B, the performance of all methods improved for estimates of both the number and locations of change points. It can be seen from Fig. 2, in the WBS method, there is a mild spread at the first location 400. MCP2 methods perform well under this model.
(d) Model D: piecewise stationary AR(1) process with a short segment
  (7) $$x_{t}=\{\begin{array}{ll} 0.75x_{t-1}+\varepsilon_{t} & 1\leq t\leq 50\\ -0.5x_{t-1}+\varepsilon_{t}, & 51\leq t\leq 1024 \end{array}$$
  LRSM remains the outstanding method in estimating the number of change points compared with the others. However, there is a large distance between estimated locations and true location in WBS and LRSM comparing with MCP2 methods, as shown in Fig. 3. MCP2 method is superior in estimating the location under this model.
(e) Model E: piecewise stationary near-unit-root process with changing variance
  (8) $$x_{t}=\{\begin{array}{ll} 0.999x_{t-1}+\varepsilon_{t} & \varepsilon_{t}\sim N(0,1),1\leq t\leq 400\\ 0.999x_{t-1}+\varepsilon_{t}, & \varepsilon_{t}\sim N\left(0,1.5^{2}\right),401\leq t\leq 750\\ 0.999x_{t-1}+\varepsilon_{t}, & \varepsilon_{t}\sim N(0,1),751\leq t\leq 1024 \end{array}$$
  Since the autocorrelation coefficients of this series remain unchanged for each segment and close to 1, all methods do not perform well.
(f) Model F: piecewise stationary AR process with high autocorrelation
  (9) $$x_{t}=\{\begin{array}{ll} 1.399x_{t-1}-0.4x_{t-2}+\varepsilon_{t} & \varepsilon_{t}\sim N\left(0,1\right),1\leq t\leq 400\\ 0.999x_{t-1}+\varepsilon_{t}, & \varepsilon_{t}\sim N\left(0,1.5^{2}\right),401\leq t\leq 750\\ 0.699x_{t-1}+0.3x_{t-2}+\varepsilon_{t}, & \varepsilon_{t}\sim N\left(0,1\right),751\leq t\leq 1024 \end{array}$$
  Simulations from models E and F are challenging data sets. From Table 3, the detection rate for all methods is quite low at around 0.3. Hence, it is not useful to plot the corresponding locations. MCP2 performs slightly better than the other two methods when the optimal scanning window is applied.
(g) Model G: piecewise stationary AR(1) process with three change points
  (10) $$x_{t}=\{\begin{array}{ll} 0.7x_{t-1}+\varepsilon_{t} & 1\leq t\leq 125\\ 0.3x_{t-1}+\varepsilon_{t} & 126\leq t\leq 532\\ 0.9x_{t-1}+\varepsilon_{t} & 533\leq t\leq 704\\ 0.1x_{t-1}+\varepsilon_{t} & 705\leq t\leq 1024 \end{array}$$
  It can be indicated from Table 3 that MCP2 outperformed the other methods under this model in terms of estimating the number of change points. Both WBS and LRSM methods suffer from the underestimation. For location estimates, there is an outlier—(*k̂*_1_ = 518, *k̂*_2_ = 704, *k̂*_3_ = 909) in Fig. 4 of MCP2BH. WBS and LRSM had similar performance. Overall, MCP2WRI is recommended for this model.
(h) Model H: piecewise stationary ARMA(1,1) process with three change points
  (11) $$x_{t}=\{\begin{array}{ll} 0.7x_{t-1}+\varepsilon_{t}+0.6\varepsilon_{t-1} & 1\leq t\leq 125\\ 0.3x_{t-1}+\varepsilon_{t}+0.3\varepsilon_{t-1} & 126\leq t\leq 532\\ 0.9x_{t-1}+\varepsilon_{t} & 533\leq t\leq 704\\ 0.1x_{t-1}+\varepsilon_{t}-0.5\varepsilon_{t-1} & 705\leq t\leq 1024 \end{array}$$
  Similar to the previous model, MCP2 has the best performance when estimating the number of change points, while the LRSM and WBS method has the tendency to underestimate the number of change points, as shown in Table 3. Furthermore, it is interesting to see that WBS and MCP2 have a mild variation at the second change-point from Fig. 5. A location estimate vector—(*k̂*_1_ = 429, *k̂*_2_ = 646, *k̂*_3_ = 705) is an outlier in WBS plot. Comparing WBS with LRSM, LRSM remains robust when estimating the locations.
(i) Model I: piecewise stationary moving average process
  (12) $$|x_{t}=\{\begin{array}{ll} \varepsilon_{t}+0.8\varepsilon_{t-1} & 1\leq t\leq 128\\ \varepsilon_{t}+1.68\varepsilon_{t-1}-0.81\varepsilon_{t-2} & 129\leq t\leq 256 \end{array}$$
Tables 1 and 3 show that all methods performed well when estimating the number of change points. In terms of estimating the locations, all methods performed poorly. Fig. 5 indicates that the estimates of LRSM and WBS method have large spread around the true change-point, while the estimates of MCP2 method tend to cluster below 128.

### 3.3 Discussion of simulation results

**Fig. 6:**
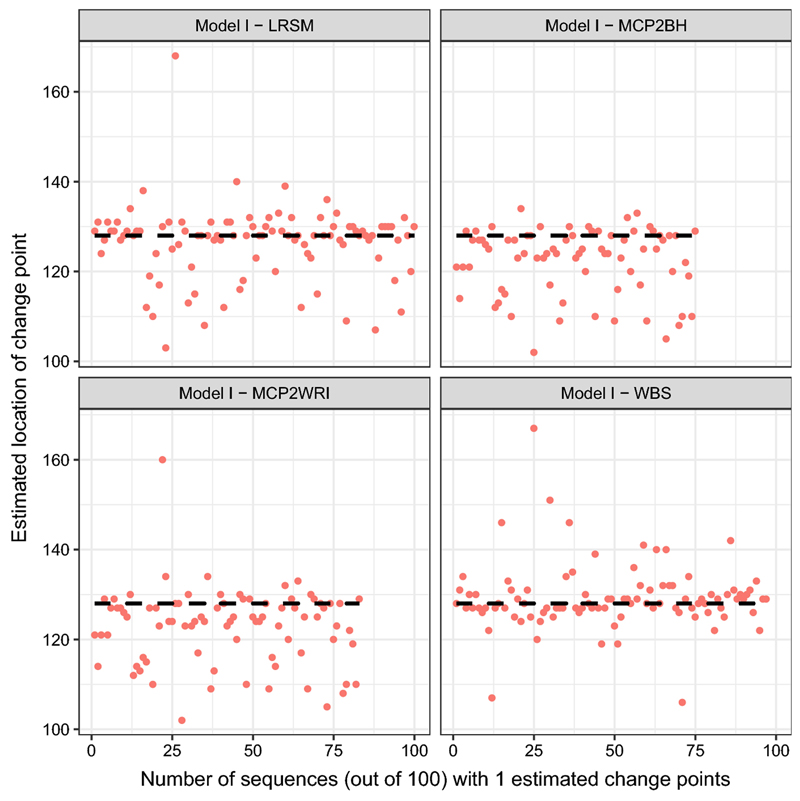
Plots of estimated locations of change points from different methods under model I. Horizontal line stands for the sequence of estimated changes only when the estimated number of change points equals to 1. The dashed black line represents the true location of change point at 128

In the simulation study, we have used nine settings to evaluate the performance of the MCP2, LRSM and WBS methods. We firstly had a discussion on the choice of scanning window. Comparing with LRSM, the implementation of MCP2 is not limited to the value of *h*. The optimal value of *h* has been provided in Table 2. Then, we evaluated the methods from two perspectives: the accuracy in detecting the number of change points and the accuracy in detecting the locations. Searching for the number of change points is the first challenge since it may be overestimated or underestimated, as shown in Table 3. We produce Figs. 1, 2, 3, 4, 5 and 6 to show that fitting between estimated change points and true change points conditioned on that the estimated number of change points is correct. Overall, the MCP2 performs well and shows its superiority under Model G. Model H and I demonstrate that detecting change-points in a piecewise stationary moving average process remains a challenge. As shown in Fig. 6, the estimates from all methods display a large spread.

## 4 Real data analysis

### 4.1 Example 1: physiological data time series

**Fig. 7:**
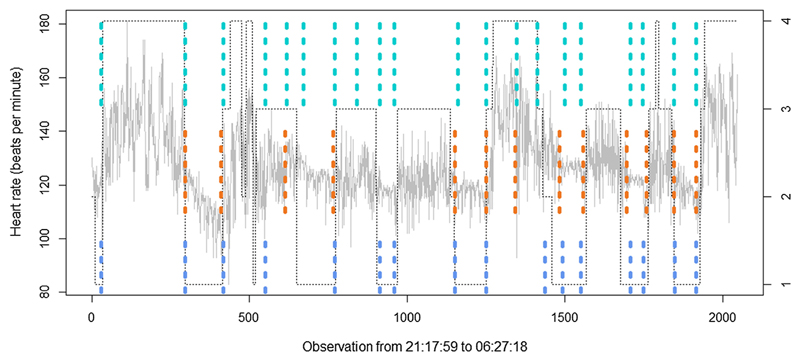
Performance of MCP2 with WBS, the top and bottom dotted line represents MCP2-BH and MCP2-Wright, the middle dotted line represents WBS method with default setting. The right hand axis represents 1 = quiet sleep, 2 = between quiet and active sleep, 3 = active sleep, 4 = awake

In this section, we use two linked medical time series, BabyECG and BabySS, which are available in the R package *wavethresh*, containing 2048 observations of an infant’s heart rate and sleep state sampled every 16 s recorded from 21:17:59 to 06:27:18. Both of them were recorded from the same 66 day old infant. The dashed line represents a change in sleep state. Korkas and Fryzlewicz (2017) has analysed the BabyECG time series as a real data example of a piecewise stationary time series by using the WBS method. Here we compare MCP2 with WBS, since LRSM is not applicable for this situation. From Fig. 7, it can be seen that all methods tend to be in agreement at most estimated change points. MCP2 is able to identify the short segment if we use the smallest scanning window whereas WBS may ignore the shorter segments. In addition, the BH procedure is more conservative than Wright’s. We remark that the selection of a scanning window exerts a control on the final estimates. In this situation, the scanning window we use is *h* = max {50, log(2048)^2^}.

### 4.2 Example 2: monthly IBM stock returns

**Fig. 8:**
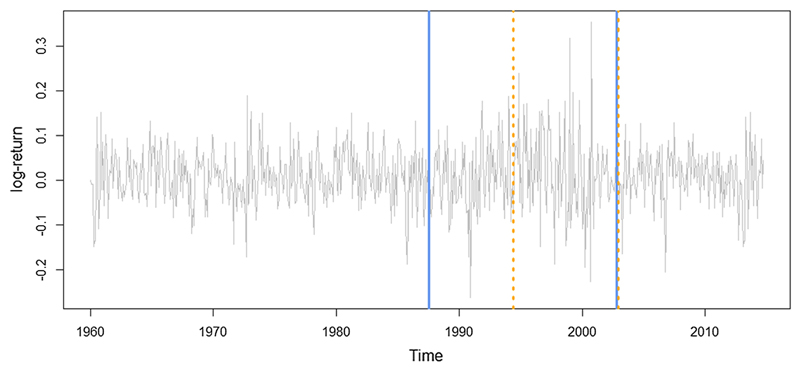
Performance of MCP2 with LRSM, the blue line represents LRSM, the orange dotted line represents MCP2-BH method

The experiment we perform here is used for comparing MCP2 with the LRSM method by analysing monthly stock returns of IBM from January 1962 to October 2014, which is an example tested by Yau and Zhao (2016) using LRSM. The scanning window used in MCP2 is the same as LRSM, which is *h* = 41. LRSM gives two changes at 307 (July 1987) and 491 (November 2002), whereas MCP2-BH gives two estimations at 390 (June 1994) and 492 (December 2002). MCP2-Wright gives only one detection at 492. It seems that there is a clear agreement on the second change point (Fig. 8).

## 5 Conclusion

In this paper, we proposed the MCP2 method which shows the flexibility and superior performance over the LRSM and WBS methods in piecewise stationary autoregressive process with more than two change points. In terms of measuring the locations of change points, we used novel statistical plots instead of the Hausdorff distance, one advantage being that we can get insights from the plots as to what caused the over-segmentation. In addition, the plots clearly demonstrated the performance of each method when estimating the locations of change points.

Although the MCP2 method worked particularly well in simulations in identifying change points when there were some, the Type I error rates were above the significance level under the null models (Model A). This may be due to the fact that, although the method accounts for multiple testing in the second (validation) stage, there is still uncertainty not accounted for from the first (detection) stage. A way of accounting for this would be to use the Bonferroni procedure with a *p*-value correction which reflects the number of scan statistics examined. A conservative approach is to set the *p*-value threshold to *α*/*T*, which will reduce the Type I error rate with the trade-off that the power to detect true change points is also reduced. Future work will refine this approach, but preliminary simulation results suggest that good power is retained compared with the other methods.

Other future research will involve a theoretical investigation of our method as well as work to further improve the estimation accuracy.

## Figures and Tables

**Table 1:** Sensitivity test of scanning window *h* for MCP2BH and MCP2WRI

MCP2WRI	*h* = 1⌊log(*T*)^2^⌋	*ml*_*k*_	% : *N̂* = *N*	$\bar{df}$	*h* = 1.5⌊log(*T*)^2^⌋	% : *N̂* = *N*	$\bar{df}$
Model A							
*β* = 0.4	48	1024	68	4.87	72	71	5.27
*β* = 0.7	48	1024	65	4.88	72	73	5.22
*β* =-0.1	48	1024	66	4.88	72	72	5.24
*β* =-0.7	48	1024	73	4.90	72	73	5.24
Model B	48	256	47	4.94	72	56	5.24
Model C	48	212	58	4.92	72	70	5.47
Model D	48	50	58	4.86	72	65	5.25
Model E	48	274	6	5.06	72	21	5.47
Model F	48	274	8	5.04	72	22	5.45
Model G	48	125	36	4.96	72	64	5.40
Model H	48	125	41	4.95	72	63	5.46
Model I	30	128	75	4.38	45	88	4.81
Model A							
*β* = 0.4	96	1024	75	5.50	120	82	5.72
*β* = 0.7	96	1024	76	5.45	120	81	5.70
*β* =-0.1	96	1024	75	5.50	120	74	5.81
*β* =-0.7	96	1024	79	5.47	120	77	5.77
Model B	96	256	70	5.69	120	87	5.86
Model C	96	212	83	5.74	120	91	5.87
Model D	96	50	70	5.45	120	78	5.62
Model E	96	274	51	5.67	120	62	5.88
Model F	96	274	36	5.74	120	41	5.91
Model G	96	125	74	5.66	120	89	5.82
Model H	96	125	74	5.61	120	60	5.70
Model I	60	128	100	5	75	100	5
Model A							
*β* = 0.4	48	1024	70	4.87	72	71	5.27
*β* = 0.7	48	1024	68	4.88	72	75	5.22
*β* =-0.1	48	1024	68	4.88	72	74	5.24
*β* =-0.7	48	1024	78	4.90	72	75	5.24
Model B	48	256	58	4.94	72	69	5.24
Model C	48	212	74	4.92	72	90	5.47
Model D	48	50	72	4.86	72	75	5.25
Model E	48	274	17	5.06	72	24	5.47
Model F	48	274	14	5.04	72	21	5.45
Model G	48	125	36	4.96	72	60	5.40
Model H	48	125	45	4.95	72	76	5.46
Model I	30	128	83	4.38	45	92	4.81
Model A							
*β* = 0.4	96	1024	76	5.50	120	84	5.72
*β* = 0.7	96	1024	77	5.45	120	81	5.70
*β* =-0.1	96	1024	76	5.50	120	76	5.81
*β* =-0.7	96	1024	80	5.47	120	78	5.77
Model B	96	256	82	5.69	120	92	5.86
Model C	96	212	95	5.74	120	94	5.87
Model D	96	50	82	5.45	120	83	5.62
Model E	96	274	58	5.67	120	65	5.88
Model F	96	274	39	5.74	120	48	5.91
Model G	96	125	68	5.66	120	79	5.82
Model H	96	125	81	5.61	120	62	5.70
Model I	60	128	100	5	75	100	5

**Table 2:** Optimal scanning window *h* selected for MCP2BH and MCP2WRI

Model	MCP2BH	MCP2WRI
	*h* = *r*⌊log(*T*)^2^⌋		
Model A		
*β* = 0.4	*r* = 2.5	*r* = 2.5
*β* = 0.7	*r* = 2.5	*r* = 2.5
*β* =-0.1	*r* = 2	*r* = 2
*β* =-0.7	*r* = 2	*r* = 2
Model B	*r* = 2.5	*r* = 2.5
Model C	*r* = 2.5	*r* = 2
Model D	*r* = 1	*r* = 1
Model E	*r* = 2.5	*r* = 2.5
Model F	*r* = 2.5	*r* = 2.5
Model G	*r* = 2.5	*r* = 2.5
Model H	*r* = 2	*r* = 2
Model I	*r* = 2	*r* = 2

**Table 3:** The simulation performance of MCP2BH, MCP2WRI, LRSM, and WBS method for estimating the number of change points

	MCP2BH			MCP2WRI			LRSM			WBS		
	*N̂*			*N̂*			*N̂*			*N̂*		
Model A	0*	1	≥ 2	0*	1	≥ 2	0*	1	≥ 2	0*	1	≥ 2
	
*β* = 0.4	0.75	0.15	0.10	0.76	0.17	0.07	1	0	0	0.93	0.06	0.01
*β* = 0.7	0.76	0.16	0.08	0.77	0.19	0.04	1	0	0	0.93	0.06	0.01
*β* = –0.1	0.75	0.13	0.12	0.76	0.19	0.05	1	0	0	0.95	0.03	0.02
*β* = –0.7	0.79	0.16	0.05	0.80	0.16	0.04	1	0	0	0.35	0.25	0.40

	0	1*	≥ 2	0	1*	≥ 2	0	1*	≥ 2	0	1*	≥ 2
	
Model D	0	0.58	0.42	0	0.72	0.28	0.03	0.97	0	0.15	0.67	0.18
Model I	0	0.75	0.25	0	0.83	0.17	0	1	0	0	0.97	0.03

	≤ 1	2*	≥ 3	≤ 1	2*	≥ 3	≤ 1	2*	≥ 3	≤ 1	2*	≥ 3
	
Model B	0	0.70	0.30	0.01	0.82	0.17	0	1	0	0.13	0.52	0.35
Model C	0	0.83	0.17	0	0.95	0.05	0	1	0	0	0.88	0.12
Model E	0.03	0.06	0.91	0.04	0.17	0.79	0.05	0.21	0.74	0.04	0.22	0.74
Model F	0	0.08	0.92	0	0.14	0.86	0.17	0.23	0.60	0.11	0.32	0.57

	≤ 2	3*	≥ 4	≤ 2	3*	≥ 4	≤ 2	3*	≥ 4	≤ 2	3*	≥ 4
	
Model G	0.09	0.74	0.17	0.22	0.68	0.10	0.60	0.40	0	0.61	0.38	0.01
Model H	0.08	0.74	0.18	0.12	0.81	0.07	0.45	0.55	0	0.46	0.54	0

The true number of change point(s) is 0^∗^, 1^∗^, 2^∗^ and 3^∗^ respectively
